# A Comprehensive Review on the Manipulation of the Sphingolipid Pathway by Pathogenic Bacteria

**DOI:** 10.3389/fcell.2019.00168

**Published:** 2019-08-21

**Authors:** Monica Rolando, Carmen Buchrieser

**Affiliations:** Biologie des Bactéries Intracellulaires, CNRS UMR 3525, Institut Pasteur, Paris, France

**Keywords:** sphingolipids, host-pathogen interactions, *Legionella*, *Pseudomonas*, *Mycobacteria*

## Abstract

Bacterial pathogens have developed many different strategies to hijack host cell responses to promote their own survival. The manipulation of lipid biogenesis and cell membrane stability is emerging as a key player in bacterial host cell control. Indeed, many bacterial pathogens such as *Legionella, Pseudomonas, Neisseria, Staphylococci, Mycobacteria, Helicobacter*, or *Clostridia* are able to manipulate and use host sphingolipids during multiple steps of the infectious process. Sphingolipids have long been considered only as structural components of cell membranes, however, it is now well known that they are also intracellular and intercellular signaling molecules that play important roles in many eukaryotic cell functions as well as in orchestrating immune responses. Furthermore, they are important to eliminate invading pathogens and play a crucial role in infectious diseases. In this review, we focus on the different strategies employed by pathogenic bacteria to hijack the sphingolipid balance in the host cell to promote cellular colonization.

## Introduction

Sphingolipids constitute an important class of lipids that are structural modules in eukaryotic membranes. However, they have also been shown to act as signaling molecules that play critical roles in regulating diverse physiological processes including signal transduction, regulation of cell growth and death, adhesion, migration, and inflammation. Indeed, sphingolipids are also bioactive molecules and their highly interconnected and spatially regulated pathways are very complex (Hannun and Obeid, 2008).

**FIGURE 1:**
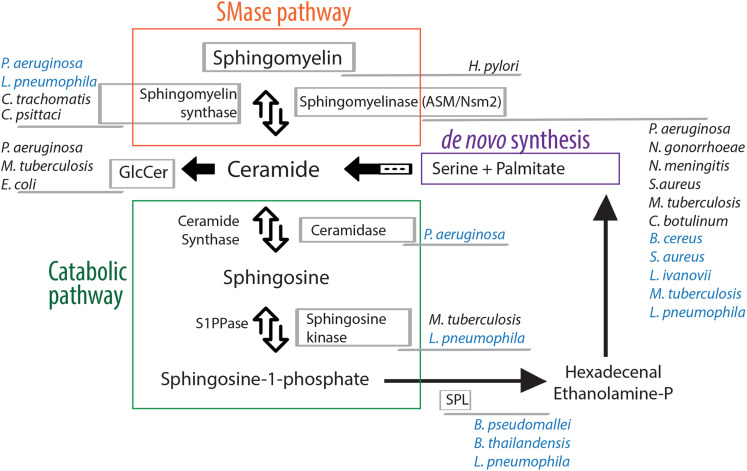
Manipulation of the sphingolipid pathway by bacterial pathogens. Ceramide, the hub of the sphingolipid pathway, can be formed by two different routes: *de novo* synthesis, that starts by the condensation of the amino acid serine and the saturated fatty acid palmitate (purple), or the sphingomyelinase pathway that allows the degradation of sphingomyelin in ceramide (orange). The catabolic pathway (green) leads to the formation of bioactive lipids via the action of different enzymes. All steps are reversible, except the sphingosine lyase activity that irreversibly cleaves sphingosine-1-phosphate to generate ethanolamine phosphate and hexadecenal, which can be subsequently reincorporated into the *de novo* synthesis. Bacterial pathogens that target host cell sphingolipid enzymes are indicated. Their names are written next to the host enzyme they target/manipulate either directly (written in blue color) or indirectly (written in black color). For a comprehensive review describing the subcellular localization of sphingolipid enzymes refer to Yamaji and Hanada (2015). SMase, sphingomyelinase; GlcCer, glucosylceramide; S1PPase, sphingosine-1-phosphate phosphatase; SPL, sphingosine-1-phosphate lyase.

Briefly, the main hub in the sphingolipid pathway is ceramide that can be synthesized *de novo* from serine and palmitate, present in the endoplasmic reticulum (ER) and in ER-associated membranes, or from the breakdown of sphingomyelin (SM) into ceramide and phosphatidylcholine catalyzed by sphingomyelinase enzymes (SMases) (Figure 1). Sphingomyelinases are classified as acidic, neutral or alkaline, based on their optimal pH and they are located in distinct cellular sub-compartments, where their products eventually mediate specific functions (Goni and Alonso, 2002). The ceramide generated by acidic SMase (ASM), for example, resides either in the lysosome or at the plasma membrane, where ceramide aggregates into microdomains. Later, aggregation of those microdomains into ceramide-enriched membrane platforms induces local changes in the membrane environment thereby affecting the permeability and the fluidity of the membrane and causing conformational changes in membrane-associated enzymes or receptors (Cremesti et al., 2002).

Ceramides themselves function also as bioactive molecules and provide a basis for the synthesis of other signaling molecules such as ceramide-1-phosphate or glucosylceramide, or they can eventually, through the catabolic pathway, be hydrolyzed by ceramidases to form sphingosine (Figure 1). Sphingosine can then be recycled into the sphingolipid pathway, the “salvage” pathway, where ceramide synthase hydrolyzes ceramide directly from sphingosine, or is phosphorylated by the sphingosines kinases (SKs). The product sphingosine-1-phosphate (S1P) can be dephosphorylated to regenerate sphingosine (through the action of specific S1P-phosphatases) or can be irreversibly cleaved by a sphingosine phosphate lyase (SPL) to generate ethanolamine phosphate and hexadecenal (which, in turn, can be reduced to palmitate and subsequently reincorporated into lipid metabolic pathways) (Figure 1). S1P is one of the most soluble sphingolipids, it is able to move between membranes, as well as act extracellularly. It interacts with sphingosine-1-phosphate receptors, S1PRs, which are high-affinity G-protein coupled receptors (Lee et al., 1998). S1PRs display selective tissue expression and activate specific intracellular signaling pathway, providing to S1P crucial roles in cell survival, cell migration and inflammation (Hla, 2004).

The sphingolipid mediators described above, play a role in many different cellular processes. For example, they modulate the reorganization of cellular membrane receptors and thus regulate the internalization of bacteria in the host cell, as well as the subsequent fusion of phagosomes and lysosomes. They are also implicated in intracellular signaling following bacterial internalization such as cytokine release, inflammatory responses and initiation of apoptosis of the infected cell (Maceyka and Spiegel, 2014). However, many bacterial pathogens have acquired the ability to counteract the cellular response and to change the sphingolipid balance of the cell they infect. The majority of these bacterial pathogens hijack different host cell factors to interfere with the sphingolipid signaling to their advantage. In contrast, a small number of them acquired the ability to produce enzymes that directly change the sphingolipid composition of host membranes in order to promote their colonization (Figure 1).

## Bacterial Pathogens Exploit and Hijack the Host Cell Sphingolipid Pathway

### Adhesion and Bacterial Uptake

The first critical step of hot-pathogen interaction is the bacteria-cell contact and eventually the entry of the pathogen into the host cell. Thus, bacterial pathogens may modulate membrane properties and signaling pathways to invade eukaryotic cells, therefore exploiting the sphingolipid pathway. In this context, one of the frequent targets of bacteria is the ASM that is known to participate in membrane reorganization and formation of ceramide-enriched platforms. Several bacterial pathogens have been shown to activate the ASM, a mechanism that promotes bacterial colonization. Furthermore, sphingosine has been shown to have antimicrobial properties as it inhibits growth and kills many Gram-positive and Gram-negative bacteria (Fischer et al., 2013). Thus decreasing sphingosine levels indirectly by activating ASM is beneficial for survival and replication of intracellular pathogens.

*Pseudomonas aeruginosa*, the primary cause of morbidity and mortality in patients with cystic fibrosis, is the bacterium for which the interaction with sphingolipids upon infection is the best studied (Teichgraber et al., 2008). In particular, *P. aeruginosa* infection triggers the activation of the ASM at the plasma membrane, with the subsequent production and release of ceramide that clusters at ceramide-enriched platforms required for bacterial internalization (Grassme et al., 2003). The increase of ceramide-enriched platforms induces a local accumulation of ß1-integrins that downregulate acid ceramidase expression, resulting in further accumulation of ceramide and consequently a reduction of surface sphingosine, a lipid that kills bacteria (Grassme et al., 2017).

Pathogenic *Neisseria* are Gram-negative pathogens that are able to bind mucosal surfaces by employing multiple strategies to interact with various cell receptors. *Neisseria gonorrhoeae*, the etiological agent of gonorrhea, and *Neisseria meningitis*, the major cause of meningitis and septicemia worldwide, are able to transiently activate the ASM to mediate the formation of ceramide-enriched platforms that favor bacterial infection. The internalization is mediated by outer membrane proteins, Opa and Opc, expressed by *N. gonorrhoeae* and *N. meningitis*, respectively. Opa is responsible for ASM activation by binding to the CEACAM receptor family (CD66) (Hauck et al., 2000), whereas Opc-expressing *N. meningitidis* induces ceramide-enriched platforms that serve to cluster the ErbB2 receptor underneath adherent bacteria (Simonis et al., 2014).

*Staphylococcus aureus*, a common commensal bacterium, but also an opportunistic pathogen that frequently causes different diseases, such as pneumonia, endocarditis, or toxic shock syndrome (Tong et al., 2015), also activates ASM. Recent studies have demonstrated that staphylococcal α-toxin is one of the factors mediating the activation of ASM and the release of ceramide via ADAM10, which is linked to the degradation of tight junctions (Becker et al., 2018). This mechanism reveals a central role for α-toxin and ASM in *S. aureus* infection, in particular in cystic fibrosis patients (Keitsch et al., 2018).

*Clostridium botulinum* C2 toxin, the binding component of the binary C2I/C2II toxin, induces a release of sphingomyelinase from lysosomes which leads to an increased level of ceramide that is responsible for the endocytosis of the toxin (Nagahama et al., 2017). Similar to *C. botulinum*, *Clostridium difficile* exploits the sphingolipid machinery to colonize the host cells as clostridium difficile toxin (CDT) causes actin ADP-ribosylation and a subsequent formation of microtubule-based membrane protrusions depending on sphingolipid-rich microdomains (Schwan et al., 2011).

The ASM is not the only sphingomyelinase playing a role in bacterial invasion as it has been shown that the neutral sphingomyelinase 2 (Nsm2) plays a role in the formation of granuloma induced by *Mycobacterium tuberculosis* in mice (Wu et al., 2018). Nsm2 is located in the inner leaflet of the plasma and Golgi membranes and has been shown to induce ceramide release upon several cellular and pathological processes (Shamseddine et al., 2015). Nsm2 is also a key factor for *N. gonorrhoeae* invasion, in particular strains expressing the major outer membrane protein PorB that binds the SREC-I receptor and triggers Nsm2 activation (Faulstich et al., 2015).

Several bacterial pathogens can directly use glycosphingolipids of the plasma membrane as receptors, in order to internalize into the target cell. In particular, lactosylceramide (LacCer) acts as a pattern recognition receptor (Nakayama et al., 2013). One example is *M. tuberculosis*, that can bind LacCer- enriched lipid rafts of human neutrophils via its membrane lipoarabinomannans (LAMs) to stimulate phagocytosis (Nakayama et al., 2016).

Glycosphingolipids are also exploited by bacterial toxins to translocate into target cells. It has been shown that globotriaosylceramide (Gb3), also known as CD77 or Pk blood group antigen, is the ligand of *Escherichia coli* shiga toxins (Lingwood et al., 2010) and lectin 1 (LecA), an outer membrane virulence factor of *P. aeruginosa*. In the case of shiga toxins, the receptor binding allows the toxin internalization and, once into the cell cytosol, the triggering of cell toxicity (Melton-Celsa, 2014), whereas LecA once it binds Gb3 triggers a signaling cascade through CrkII phosphorylation (Zheng et al., 2017). This interaction also promotes a cell membrane engulfment, that prompts *P. aeruginosa* uptake by host cells (Eierhoff et al., 2014).

Sphingomyelin is also required for entry of *Helicobacter pylori*, a gastric pathogen causing chronic infections that are a significant risk factor for the development of ulcer disease or gastric adenocarcinoma in epithelial cells. The secreted vacuolating cytotoxin (VacA) plays an important role in bacterial colonization and multiple putative VacA receptors have been reported (Foegeding et al., 2016). Between them sphingomyelin is essential for targeting VacA to membrane rafts and subsequent Cdc42-dependent pinocytic cellular entry (Gupta et al., 2010).

### Phagolysosome Fusion and Formation of Intracellular Compartments for Bacterial Replication

An efficient host response to bacterial invasion consists in an appropriate fusion between phagosomes and lysosomes carrying the pathogens to elimination. Interestingly sphingolipids, and in particular ASM, play a role in mediating phagolysosome fusion and degradation of bacteria. In fact, a high susceptibility to bacterial infection of *Listeria monocytogenes* and *S. aureus* has been shown in ASM deficient models. The role of ASM in *L. monocytogenes* uptake and invasion has been shown both in cellular (macrophages) (Schramm et al., 2008), and in animal models (mice) (Utermöhlen et al., 2003).

*Staphylococcus aureus* infection causes the activation of CD44 receptor, which is stimulating ASM via the generation of reactive oxygen species (ROS), resulting in ceramide release and increased formation of ceramide-enriched domains after infection. These domains cluster and thereby amplify CD44 signaling resulting in further activation of the ASM providing a positive forward feedback loop between CD44 and the ASM. CD44 activation by *S. aureus* stimulates small G proteins, a reorganization of the cytoskeleton, internalization of the pathogen, and fusion of phagosomes with lysosomes, a process that requires again ASM (Li et al., 2017).

*Mycobacterium tuberculosis* is able to actively inhibit phagosome maturation by acting on sphingosine kinase 1. This pathogen inhibits both, the activation and the translocation of SK1 to block the cytosolic Ca^2+^ signaling, required for normal maturation of phagosomes (Thompson et al., 2005). In contrast to *M. tuberculosis, Chlamydia trachomatis*, a Gram-negative obligate intracellular pathogen responsible for trachoma and sexually transmitted diseases, develops, after binding and entry into target cells, a membrane-bound vacuole, termed inclusion that minimizes the interaction with immune defenses and other host-derived molecules. It has been shown that the inclusion membrane contains sphingomyelin (Hackstadt et al., 1996) and that *C. trochomatis* and *Chlamydia psittaci* actively redirect sphingomyelin biosynthesis at the inclusion membrane by recruitment of sphingomyelin synthases, a step strictly necessary for inclusion growth and stability (Elwell et al., 2011; Koch-Edelmann et al., 2017).

### Signal Transduction, Apoptosis, and Autophagy

Sphingolipid turnover affects the intracellular trafficking of important membrane microdomains, impacting their associated receptors, transporters and the production of a cascade of products, each of which can interact with multiple intracellular targets (Ohanian and Ohanian, 2001). Several pathogens are able to modulate the cellular transduction during infection upon a direct targeting of sphingolipid enzymes. One example is the signaling pathway activated by sphingosine kinase 1 upon *Mycobacterium smegmatis* infection. Prakash et al. (2010) showed that sphingosine kinase 1 inhibition in infected macrophages leads to a decrease in anti-mycobacterial proteins-pp38 and iNOS, via dampened NF-kB and p38-MAPK activities. In a similar manner, specific activation of mitochondrial ASM by *P. aeruginosa* triggers the release of mitochondrial ceramide and the release of cytochrome-c from mitochondria, leading to cell death (Manago et al., 2015). Possibly this apoptotic process is mediated by the formation of ceramide channels in the mitochondrial outer membrane through which cytochrome c can exit mitochondria and activate apoptotic pathway (Ganesan et al., 2010).

Sphingolipids are well known in mediating key cellular processes, including autophagy (Bedia et al., 2011; Jiang and Ogretmen, 2014). *Salmonella* spp. is a food-borne Gram-negative entero-pathogen that remains a major public health problem word wide. After internalization, a type-III secretion system (T3SS) is necessary to remodel the phagosome into a *Salmonella* containing vacuole (SCV) where the bacterium replicates. *Salmonella*, depending on the stage of replication, can induce a suppression of autophagy, in order to enhance bacterial survival (Owen et al., 2014). This induction seems to be orchestrated by sphingolipid biomolecules, as inhibition of *de novo* sphingolipid synthesis leads to decreased *Salmonella*-induced autophagy (Huang, 2016).

## Bacterial Pathogens Mimic Host Sphingolipid Enzymes

While most bacteria do not contain sphingolipids, some of them have evolved mechanisms by which they can utilize sphingolipids of the eukaryotic cells. Interestingly, certain bacterial pathogens encode enzymes implicated in the catabolic pathway of sphingolipids in the eukaryotic cells (Figure 1 and Table 1).

**TABLE 1:** Bacterial enzymes that mimic host sphingolipids.

**Bacterium**	**Enzyme**	**Gene**	**Protein**	**References**
*B. cereus*	Sphingomyelinase	*sph*	*Bc*-SMase	Oda et al., 2012
*S. aureus*	Sphingomyelinase	*hlb*	ß-toxin	Herrera et al., 2017
*L. ivanovii*	Sphingomyelinase	*smcL*	SmcL	Gonzalez-Zorn et al., 1999
*M. tuberculosis*	Sphingomyelinase	*rv0888*	SpmT	Speer et al., 2015
*L. pneumophila* strain Paris	Sphingomyelinase^∗^	*lpp2641*	*–*	Cazalet et al., 2004
*L. pneumophila* strain Longbeachae	Sphingomyelinase^∗^	*llo2622 llo1999 llo1141*	–	Cazalet et al., 2004
*P. aeuroginosa*	Sphingomyelin synthase	*PlcH*	PlcH	Luberto et al., 2003
*P. aeuroginosa* strain AN17	Ceramidase	*PA0845*	PaCD	Okino et al., 1998
*L. pneumophila* strain Paris	Sphingosine kinase^∗^	*lpp2295*	–	Cazalet et al., 2004
*B. pseudomallei* strain K96243	Sphingosine phosphate lyase	*BPSS2021 BPSS2025*	BPSS2021 BPSS2025	Custodio et al., 2016
*B. thailandensis* strain E264	Sphingosine phosphate lyase	*BTH_II0309 BTH_II0311*	BTH_II0309 BTH_II0311	Custodio et al., 2016
*L. pneumophila* strain Paris	Sphingosine phosphate lyase^∗^	*lpp2128*	LpSPL	Cazalet et al., 2004; Rolando et al., 2016a

*^∗^The functional annotation is based on sequence similarity.*

Examples are *Bacillus cereus*, a facultative anaerobic Gram-positive bacterium associated with food poisoning and nosocomial infections, *S. aureus* a Gram-positive facultative pathogen and *Listeria Ivanovii*, a ruminant pathogen. All three encode enzymes with a high degree of amino acid sequence homology that encode sphingomyelinase (Smase) activities. *S. aureus* ß-toxin confers to the bacterium its hemolytic and lympholytic activities (Herrera et al., 2017), whereas *Bc*-Smase, produced in large amounts by clinical isolates of *B. cereus*, enhances bacterial colonization by inducing clustering of ceramide and attenuation of membrane fluidity (Oda et al., 2012). *L. ivanovii* Smase, encoded by the *smcL* gene induces hemolysis and facilitates the disruption of the phagocytic vacuole thereby promoting intracellular survival and propagation (Gonzalez-Zorn et al., 1999).

*Pseudomonas* species are also known to express and secrete sphingolipid-metabolizing enzymes. *P. aeruginosa* encodes a sphingomyelin synthase, PlcH, which specifically recognizes the choline head-group of sphingomyelin as well as the primary hydroxyl group of ceramide (Luberto et al., 2003) and its gene expression is strictly regulated by cellular amounts of sphingolipids (Okino and Ito, 2016). Furthermore, *P. aeruginosa* seems also to be able to hydrolyze ceramide as an alkaline ceramidase (Cdase) has been characterized from the *P. aeruginosa* strain AN17 isolated from the skin of a patient with atopic dermatitis (Okino et al., 1998). Enzymatic characterization of the *P. aeruginosa* Cdase showed that it can be inhibited by sphingosine and by sphingosine analogs, but not by typical mammalian Cdase inhibitors. This suggests that the bacterial Cdase has a novel active site and/or substrate-binding region (Nieuwenhuizen et al., 2002).

*Mycobacterium tuberculosis* encodes a sphingomyelinase, SpmT, that is a cell-surface exposed protein, anchored in the outer membrane, that possesses a strong sphingomyelinase activity which is required for bacterial growth and nutrient acquisition (Speer et al., 2015). The hydrolyzed products, ceramide and phosphocholine, are utilized by *M. tuberculosis* as source of carbon, nitrogen and phosphorous, respectively, explaining the stimulating activity of sphingomyelin on bacterial growth described in the past (Dubos, 1948).

*Burkholderia pseudomallei* and *Burkholderia thailandensis*, two closely related intracellular Gram-negative pathogens found in soils and water, encode SPL like proteins (Custodio et al., 2016). *B. pseudomallei*, the causative agent of melioidosis, is able to invade, survive and replicate in both phagocytic and non-phagocytic cells, whilst *B. thailandensis*, although it displays a similar intracellular phenotype, exhibits an attenuated form of the disease (Lennings et al., 2018). Custodio et al. (2016) showed that orthologs *Burkholderia* SPL proteins possess SPL activity and that they play a critical role in virulence. In addition, treatment of *Burkholderia*-infected macrophages with exogenous SPL-receptor agonists enhances their bactericidal activity (Custodio et al., 2016).

A striking example of a bacterial pathogen encoding sphingolipid enzymes is *Legionella pneumophila*, a Gram-negative intracellular bacterium responsible for Legionnaire’ disease, a severe pneumonia that is often fatal when not treated rapidly (Steinert et al., 2007). Shortly after its discovery in 1977, it has been shown that *L. pneumophila* is pathogenic for freshwater and soil amoebae (Rowbotham, 1980), leading to the new perception in microbiology, whereby bacteria that parasitize protozoa can utilize similar processes to infect human cells (Escoll et al., 2013). Genome analyses uncovered that the ability of *Legionella* to infect eukaryotic cell is partly due to the acquisition of eukaryotic gene functions from their protozoan hosts due to the *Legionella*-protozoa coevolution (Nora et al., 2009; Gomez-Valero and Buchrieser, 2013). Interestingly, *L. pneumophila* has been shown to encode for at least three proteins mimicking the host sphingolipid pathway (Cazalet et al., 2004): a sphingomyelinase, a sphingosine kinase and a sphingosine-1-phosphate lyase (Rolando et al., 2016a).

Till now only the sphingosine-1-phosphate lyase has been characterized functionally (Khweek et al., 2013; Rolando et al., 2016b). Indeed, *Legionella* SPL is encoded by all *L. pneumophila* strains analyzed, but *Legionella Longbeachae*, and is highly homologous to the eukaryotic SPL (Gomez-Valero and Buchrieser, 2019). The secreted protein effector (named *Lp*Spl and LegS2 in *L. pneumophila* strains Paris and Philadelphia, respectively) possesses SPL activity and triggers the reduction of several sphingolipids in infected host cells. Thus, *Lp*SPL alone is sufficient to prevent an increase in sphingosine levels in infected cells in order to inhibit autophagy during infection (Rolando et al., 2016b). This strategy allows the bacterium to counteract the host cell response and to facilitate intracellular growth.

## Conclusion and Outlook

Several bacterial pathogens have been shown to actively modulate the sphingolipid pathway of their host cells to promote cellular colonization. Among the different strategies employed one of the commonly targeted activity is that of the acid sphingomyelinase (ASM), which can be regulated by bacterial virulence factors. ASM activation leads to an increase of the membrane levels of ceramide resulting in the formation of ceramide-enriched membrane platforms. These structures form a unique microenvironment with biophysical properties that allow them to trap and cluster receptor molecules and intracellular signaling molecules, thereby permitting and amplifying signal transduction. Thus, ceramide acts by re-organizing molecules in cells and in that way bacteria can regulate their internalization in the host cell, the subsequent cytokine release and inflammatory response or the regulation of cell death (Gulbins et al., 2004). This specific ASM activation is driven not only by secreted virulence factors and toxins, but also by the bacterial lippopolysaccaride (LPS) itself. Indeed, the exposure of diverse cell types to LPS induces an activation of ASM and a release of ceramide.

Sphingolipids play also an important role in respiratory tract infections, as they are one of the active constituents of the mucus secreted by the alveolar epithelium, which protects the lung tissue from invading pathogens. A large number of intermediate metabolites in the mucus are secreted by the alveolar epithelium where they act as surfactants and maintain the barrier integrity. Thus, the sphingolipid balance plays an additional role in lung infection diseases. Sphingolipids, in particular ceramide and sphingosine, are in particular important in lung antibacterial defense (Seitz et al., 2015). It is thought, that in healthy individuals the constitutive presence of sphingosine in upper airway cells helps to eliminate pathogens that become highly infective in diseased lungs, e.g., cystic fibrosis, where the concentrations of both sphingosine and ceramide are altered (downregulated and upregulated, respectively). Indeed, the normalization of the lipid levels in a mouse model of cystic fibrosis was shown to be sufficient to prevent infections (Pewzner-Jung et al., 2014).

These observations point to a possible antibacterial effect of sphingolipids which could perhaps be exploited in times where antibiotic resistance has become a severe threat to global public health and it has become highly important to identify novel therapeutic targets to fight bacterial infections. Antibacterial activity of diverse sphingolipids has been shown in several types of bacterial infections and thus they are a potential new tool to fight them (Baker et al., 2018).

Actually, sphingosine has been shown to prevent *P. aeuroginosa* and *S. aureus* infections in mice (Pewzner-Jung et al., 2014; Tavakoli Tabazavareh et al., 2016). At present it is unknown how it is able to kill pathogens, however, recent findings suggest that it can cause ultrastructural damages, both extracellularly and intracellularly (Fischer et al., 2013). Resistant *S. aureus* strains, in particular methicillin-resistant *S. aureus* strains, have become an important clinical problem and are recognized as serious threats in communities and hospitals worldwide (Grundmann et al., 2006). It is thus crucial to find new therapeutic strategies and to provide alternatives to existing approaches. One possibility could be the combination of antibiotics with new target drugs as, for example, a specific inhibitor of the sphingolipid catabolic pathway. Peng et al. (2015) showed that ASM inhibition successfully rescues mice from the lethality of *S. aureus* infection.

It has also been suggested that sphingosine possesses an anti-biofilm activity by inhibiting bacterial adherence of *Streptococcus mutans*, a highly cariogenic bacterium (Cukkemane et al., 2015). An antibacterial activity has been shown also for ceramide, that has been proven to actively kill pathogenic *Neisseriae*, likely by causing dissipation of the membrane potential (Becam et al., 2017). In addition to ceramide and sphingosine, other sphingolipid metabolites, in particular S1P may offer therapeutic benefits when managing bacterial diseases. S1P has been shown to increase intracellular killing of *M. tuberculosis* by macrophages (Garg et al., 2004), as well as to reduce neonatal death associated with pertussis infections (Scanlon et al., 2015). As sphingolipids play important roles in controlling infection, future research to get a deeper insight in their functioning and the different signaling roles might allow to develop new strategies to fight bacterial pathogens. However, it would also be very interesting to study in depth how bacterial pathogens may exploit sphingolipids to their own advantage. Indeed, as discussed in this review, several intracellular pathogens that live in close contact with eukaryotic hosts have evolved strategies allowing them to mimic their functions and thereby to promote their intracellular replication. One example is *L. pneumophila*, a bacterium that has acquired several eukaryotic-like proteins in its effector arsenal among which are three enzymes that share activities of eukaryotic enzymes that act in the sphingolipid degradation pathway. However, many other intracellular pathogens, such as *Coxiella*, *Brucella*, or *Chlamydia* may encode among the many effectors for which the functions are not known yet, effectors mimicking or targeting the sphingolipid pathway. Their identification and characterization would help to not only better understand the bacterial strategy, but probably also new cellular pathways regulated by the sphingolipid bioactive molecules.

## Author Contributions

Both authors listed have made a substantial, direct and intellectual contribution to the work, and approved it for publication.

## Conflict of Interest Statement

The authors declare that the research was conducted in the absence of any commercial or financial relationships that could be construed as a potential conflict of interest.
