# Corrigendum: A Comprehensive Review on the Manipulation of the Sphingolipid Pathway by Pathogenic Bacteria

**DOI:** 10.3389/fcell.2021.647045

**Published:** 2021-02-03

**Authors:** Monica Rolando, Carmen Buchrieser

**Affiliations:** Biologie des Bactéries Intracellulaires, CNRS UMR 3525, Institut Pasteur, Paris, France

**Keywords:** sphingolipids, host-pathogen interactions, *Legionella*, *Pseudomonas*, *Mycobacteria*

There is an error in the Funding statement. The correct number for **ANR-18-CE18-0005-01** is **ANR-18-CE15-0005-01**.

The authors apologize for this error and state that this does not change the scientific conclusions of the article in any way. The original article has been updated.

